# Patterns and potential indications of WHO AWaRe Watch antibiotic use across UK community and hospital care: a national observational study

**DOI:** 10.1093/eurpub/ckag198

**Published:** 2026-11-03

**Authors:** Elin J Rowlands, Cecilia Campanile, Hiba Junaid, Jennifer C E Lane, Usama Rahman, Ben Eaton, Xavier L Griffin, Andy South, Ana Cavalcante, Duncan Cartner, Steve Harris, Ofran Almossawi, Lydia Briggs, Daniel Key, Prasiddha Khadka, Timothy Howcroft, Vishnu Vardhan Chandrabalan, Peter S Hall, Mahéva Vallet, Marta Pasikowska, Nicola Symmers, Colin McLean, Spyro Nita, Daniel Dedman, Zara Cuccu, Stelios Theophanous, Geoff Hall, Martí Català, Daniel Prieto Alhambra, Edward Burn, Danielle Newby

**Affiliations:** Health Data Sciences (HDS), Translational Sciences, Botnar Research Centre, University of Oxford, Oxford, United Kingdom; Health Data Sciences (HDS), Translational Sciences, Botnar Research Centre, University of Oxford, Oxford, United Kingdom; Bone and Joint Health, Queen Mary University of London, London, United Kingdom; Barts Health NHS Trust, NHS Trust, London, United Kingdom; Barts Life Sciences, Queen Mary University of London, London, United Kingdom; Bone and Joint Health, Queen Mary University of London, London, United Kingdom; Barts Health NHS Trust, NHS Trust, London, United Kingdom; Bone and Joint Health, Queen Mary University of London, London, United Kingdom; Barts Health NHS Trust, NHS Trust, London, United Kingdom; Bone and Joint Health, Queen Mary University of London, London, United Kingdom; Barts Health NHS Trust, NHS Trust, London, United Kingdom; Bone and Joint Health, Queen Mary University of London, London, United Kingdom; Barts Health NHS Trust, NHS Trust, London, United Kingdom; NIHR University College London Hospitals Biomedical Research Centre, London, United Kingdom; NIHR University College London Hospitals Biomedical Research Centre, London, United Kingdom; NIHR University College London Hospitals Biomedical Research Centre, London, United Kingdom; NIHR University College London Hospitals Biomedical Research Centre, London, United Kingdom; Great Ormond Street Hospital for Children, NHS Foundation Trust, London, United Kingdom; Great Ormond Street Hospital for Children, NHS Foundation Trust, London, United Kingdom; Great Ormond Street Hospital for Children, NHS Foundation Trust, London, United Kingdom; Great Ormond Street Hospital for Children, NHS Foundation Trust, London, United Kingdom; Lancashire Teaching Hospitals, NHS Foundation Trust, Preston, United Kingdom; Lancashire Teaching Hospitals, NHS Foundation Trust, Preston, United Kingdom; Faculty of Health and Medicine, Lancaster University, Lancaster, United Kingdom; DataLoch, NHS Lothian, Edinburgh, United Kingdom; DataLoch, NHS Lothian, Edinburgh, United Kingdom; DataLoch, NHS Lothian, Edinburgh, United Kingdom; DataLoch, NHS Lothian, Edinburgh, United Kingdom; DataLoch, NHS Lothian, Edinburgh, United Kingdom; DataLoch, NHS Lothian, Edinburgh, United Kingdom; Clinical Practice Research Datalink (CPRD), Medicines and Healthcare products Regulatory Agency (MHRA), London, United Kingdom; Clinical Practice Research Datalink (CPRD), Medicines and Healthcare products Regulatory Agency (MHRA), London, United Kingdom; Leeds Teaching Hospitals, NHS Trust, Leeds, United Kingdom; Leeds Institute of Medical Research, University of Leeds, Leeds, United Kingdom; Health Data Sciences (HDS), Translational Sciences, Botnar Research Centre, University of Oxford, Oxford, United Kingdom; Health Data Sciences (HDS), Translational Sciences, Botnar Research Centre, University of Oxford, Oxford, United Kingdom; Department of Medical Informatics, Erasmus MC University, Rotterdam, The Netherlands; Health Data Sciences (HDS), Translational Sciences, Botnar Research Centre, University of Oxford, Oxford, United Kingdom; Health Data Sciences (HDS), Translational Sciences, Botnar Research Centre, University of Oxford, Oxford, United Kingdom

## Abstract

There are major concerns regarding the risk of antimicrobial resistance evoked through the inappropriate use of antibiotics. The WHO AWaRe classification has identified antibiotics that have high resistance potential. This cohort study utilized data from seven UK databases, including two community care databases and five hospital databases. The study period was from 2012 to 2025, or from the start of electronic prescription data. Longitudinal trends in Watch antibiotic use and patient demographic information were summarized. Data-driven large-scale characterization was used to identify conditions of interest, and prior use of Access and other Watch antibiotics was summarized. The study included 433 368 hospital and 11 705 843 primary care records of Watch antibiotic use. Quarterly crude incidence rates showed seasonal variation in community prescribing, with rates generally higher in the third and fourth quarters. Although use in community care increased during the COVID-19 pandemic, it remained below prepandemic levels. Most community users were female, while most hospital users were male. Hospital users had more severe conditions, including healthcare-associated infections and sepsis, while community care users were more commonly treated for skin and respiratory tract infections. Prior Access antibiotic use was generally recorded in fewer than 50% of patients. Prior Access of Watch antibiotic use was minimal in community care. Our results highlight the complexity of antibiotic use across different healthcare settings. Regional and demographic variation indicates areas where prescribing practices could be refined. Ongoing surveillance and targeted stewardship interventions are essential to promote appropriate use and prevent resistance.

## Introduction

Bacterial infections are a major cause of morbidity and mortality worldwide [1]. Antibiotics have been hugely successful in improving health outcomes and have significantly contributed to the global reduction in mortality in the under 5’s [2]. While essential for treating bacterial infections, antibiotics pose a major public health challenge when misused, as this can accelerate the development of antimicrobial resistance (AMR) [3]. AMR makes infections more difficult to treat and increases risks in medical procedures such as surgery and chemotherapy [4]. In 2021, bacterial AMR was linked to 4.71 million deaths globally, including 1.14 million deaths directly attributed to resistant infections [5].

To help prevent AMR, the World Health Organization (WHO) introduced the AWaRe classification of antibiotics in 2017 as part of its global stewardship strategy. This classification categorized antibiotics into three groups: Access (low resistance potential), Watch (high resistance potential), and Reserve (last resort options) [6]. The National Institute for Health and Care Excellence (NICE) guidelines generally recommend Access antibiotics as the first-line treatment for certain conditions, while Watch antibiotics are commonly recommended as alternative first-line therapy or second-line therapy for common conditions such as urinary tract and respiratory tract infections [7, 8]. However, rarer and more complex infections often require Watch antibiotics as first-line therapy, e.g. *Clostridioides difficile* [9].

The use of Watch antibiotics should be carefully managed, as inappropriate use such as administering antibiotics without a clear clinical indication or without prior use of recommended first-line Access or Watch group antibiotics (if applicable) can contribute to the development and spread of AMR [10]. However, little is known about the utilization of Watch antibiotics in the United Kingdom (UK). Careful management of Watch antibiotic use is needed to ensure they remain available when clinically necessary, while minimizing exposure to prevent further resistance.

In this study, we aimed to better understand the trends, characteristics, and conditions of interest associated with Watch antibiotic use across community and hospital care in the UK. By providing insight into trends of Watch antibiotic use, our findings can support targeted stewardship strategies and inform policies to help mitigate the development and spread of AMR.

## Methods

### Study design

We conducted a network cohort study across the UK using routinely collected healthcare data mapped to the Observational Medical Outcomes Partnership Common Data Model (OMOP CDM) [11]. The OMOP CDM allowed the study to be run by each site with common analytical code. Results were aggregated without sharing individual patient-level data.

### Data sources

The study was performed across the recently established Health Data Research OMOP Network (HERON), which to date includes seven data partners covering different healthcare settings and geographies all mapped to the OMOP CDM [12]. Five hospital databases were included: Barts Health NHS Trust (Barts), Great Ormond Street Hospital NHS Foundation Trust (GOSH), Lancashire Teaching Hospitals (IDRIL), Leeds Teaching Hospitals NHS Trust (LTHT), and University College London Hospitals NHS Foundation Trust (UCLH), as well as two databases with community care data: the UK community care Clinical Practice Research Datalink (CPRD) Aurum and NHS Lothian (DataLoch).

CPRD Aurum includes data from England, whereas DataLoch covers the Lothian region of Scotland. GOSH is a tertiary and quaternary specialist paediatric hospital serving patients from across the UK and internationally. Barts, LTHT, UCLH, and IDRIL are large NHS teaching hospital trusts providing a broad range of secondary, tertiary, and specialist care. Full descriptions of each database are available in Table S1.

### Study participants

The study period was from the start of usable data to the end of database capture. For CPRD Aurum and DataLoch, usable data were available from 2012, while for the remaining data partners, this was from 2022. In the community care databases (CPRD Aurum and DataLoch), individuals were considered under observation from their first record (equivalent to general practitioner (GP) registration in CPRD) to deregistration, death, or end of database capture. In hospital databases, individuals were under observation from the first recorded visit, condition, or drug exposure. Observation continued for up to 18 months following the most recent record. Overlapping observations were merged into a single observation episode. Observation stopped at death or end of database. In GOSH, individuals were no longer considered under observation once they reached 18 years of age. For hospital databases, only Watch antibiotics used during an inpatient or Accident & Emergency (A&E) visit were included to capture Watch antibiotic use during acute hospital care.

### Primary outcomes

The main outcome of interest was prescription/dispensation of each antibiotic from the Watch category of the WHO AWaRe classification (2023). All drug concepts that included a Watch antibiotic, whether alone or in combination with other active ingredients, were included. Only antibiotics with at least 100 patient records during the study period were included. In total, we included 134 Watch antibiotics and 72 Access antibiotics (Tables S2 and S3).

Individuals could contribute multiple records of Watch antibiotic use, provided there was a wash-out period of at least 30 days between exposures. Records within 7 days of each other were merged.

### Statistical methods

Descriptive statistics were used to summarize characteristics for patient records for each Watch antibiotic for each database. Use of Watch and Access antibiotics prior to initiation of each Watch antibiotic and conditions of interest recorded in the 14 days before and after initiation were also summarized. Median (IQR) and mean (SD) were used for continuous variables and counts and percentages used for categorical variables. Characterization was performed using the CohortCharacteristics R package [13].

Information on the clinical indication for antibiotic prescribing or dispensing was not available in the databases included in this study. Therefore, conditions of interest were identified by a clinician and comprised a broad range of infections, including cardiac, ear, eye, gastrointestinal, respiratory tract, skin, and urinary tract infections. Additional categories included conditions associated with an increased risk of infection [exacerbation of chronic obstructive pulmonary disease (COPD), exacerbation of cystic fibrosis, neutropenia, and agranulocytosis], healthcare-associated infections (e.g. surgical site infections), infections caused by antimicrobial-resistant bacteria, sepsis, and infections where the site was unspecified (‘other’). We also included a category of signs and symptoms commonly associated with infection, such as fever, nausea, and cough. A full list of the conditions along with the concepts included is available in Table S4.

Overall and quarterly crude incidence rates (IRs) for each Watch antibiotic were calculated from 2022 to the end of available data for each database. For incidence, the number of events, the observed time at risk, and the IR per 100 000 person-years were summarized along with 95% confidence intervals (95% CI). We calculated 95% CI of the estimated rates using the Poisson distribution. We allowed for repetitive events. IRs were calculated overall and stratified by age group and sex. Incidence was estimated using the IncidencePrevalence R package [14].

Counts smaller than five were suppressed across all databases to prevent re-identification. For LTHT, counts smaller than six were suppressed, and for DataLoch, counts smaller than 10 were suppressed. All analytical code is available in a public GitHub repository: https://github.com/heron-uk/HDRUK-01-001-Antibiotics.

### Patient and public involvement

This research question was chosen in collaboration with key national stakeholders, including HDRUK, HDR Alliance, the NHS, NICE, MHRA, and UKHSA including patients and the public.

### Ethics

This work uses data provided by patients and collected by the NHS as part of their care and support. Informed consent of individual patients was not required as anonymized information was obtained from medical records.

## Results

### Overview

A total of 433 368 and 13 423 216 records of Watch antibiotic use were found in hospital and community care settings, respectively. Across all databases, we had results for 33 different Watch antibiotics. All study results can be found in the accompanying Shiny app (https://dpa-pde-oxford.shinyapps.io/heronAntibioticsStudy/).

In CPRD Aurum, the most used Watch antibiotic between 2012 and 2025 was neomycin with 4 477 598 records (Table 1), and in DataLoch, it was clarithromycin, with 394 394 records. The combination of piperacillin and tazobactam was the most frequently used Watch antibiotic in Barts (*n* = 19 976), LTHT (26 365), and GOSH (4227) between 2022 and 2025, while cefuroxime was most frequent in IDRIL (10 801) and UCLH (44 228) (Table 2). As the results for piperacillin and tazobactam were nearly identical, only the results for piperacillin are presented, but it is labelled as piperacillin/tazobactam. Patient demographics for the top five Watch antibiotics for each database, including age and sex, can be found in Tables S5–S11.

**Table 1:** Five most commonly used Watch antibiotics, ranked by number of records (*n*), in each database.^a^

Rank	CPRD Aurum	Barts	DataLoch	GOSH	IDRIL	LTHT	UCLH
1	Neomycin (4 477 598)	**Piperacillin/tazobactam (19** **976)**	**Clarithromycin (394** **394)**	**Piperacillin/tazobactam (4227)**	Cefuroxime (10 801)	**Piperacillin/tazobactam (26** **365)**	Cefuroxime (44 228)
2	Fusidate (2 029 304)	**Clarithromycin (11** **794)**	Fusidate (390 456)	Teicoplanin (2025)	**Clarithromycin (9870)**	**Clarithromycin (11** **567)**	**Ciprofloxacin (19** **251)**
3	**Ciprofloxacin (1** **561** **067)**	**Ciprofloxacin (11** **359)**	Neomycin (353 001)	Vancomycin (1757)	**Piperacillin/tazobactam (6642)**	Cefuroxime (11 270)	**Piperacillin/tazobactam** **(10** **450)**
4	Erythromycin (1 295 225)	Ceftriaxone (10 841)	Oxytetracycline (178 584)	**Ciprofloxacin (1571)**	**Ciprofloxacin (2400)**	**Ciprofloxacin (10** **043)**	**Clarithromycin (9842)**
5	Clarithromycin (769 070)	Meropenem (7417)	Erythromycin (136 037)	Meropenem (1527)	Levofloxacin (2314)	Teicoplanin (9608)	Teicoplanin (5547)

a Antibiotics in top five among at least five databases are shown in bold.

### Incidence rates

In hospital care, IR trends for the most common Watch antibiotics varied across sites (Fig. 1). For example, piperacillin/tazobactam use remained relatively stable across all hospitals, with overall IR ranging from 191 (95% CI 187–196) to 4494 (4440–4549) per 100 000 person-years in IDRIL and LTHT, respectively; however, GOSH showed a decline from 1786 (1601–1987) to 1349 (1198–1514) between Q1 2022 and Q4 2023, before increasing to 1495 (1339–1663) in Q4 2024. Other notable trends include an increase in cefuroxime use in Barts, LTHT, UCLH, and IDRIL over the study period, while a gradual decrease was observed in GOSH.

**Figure 1:**
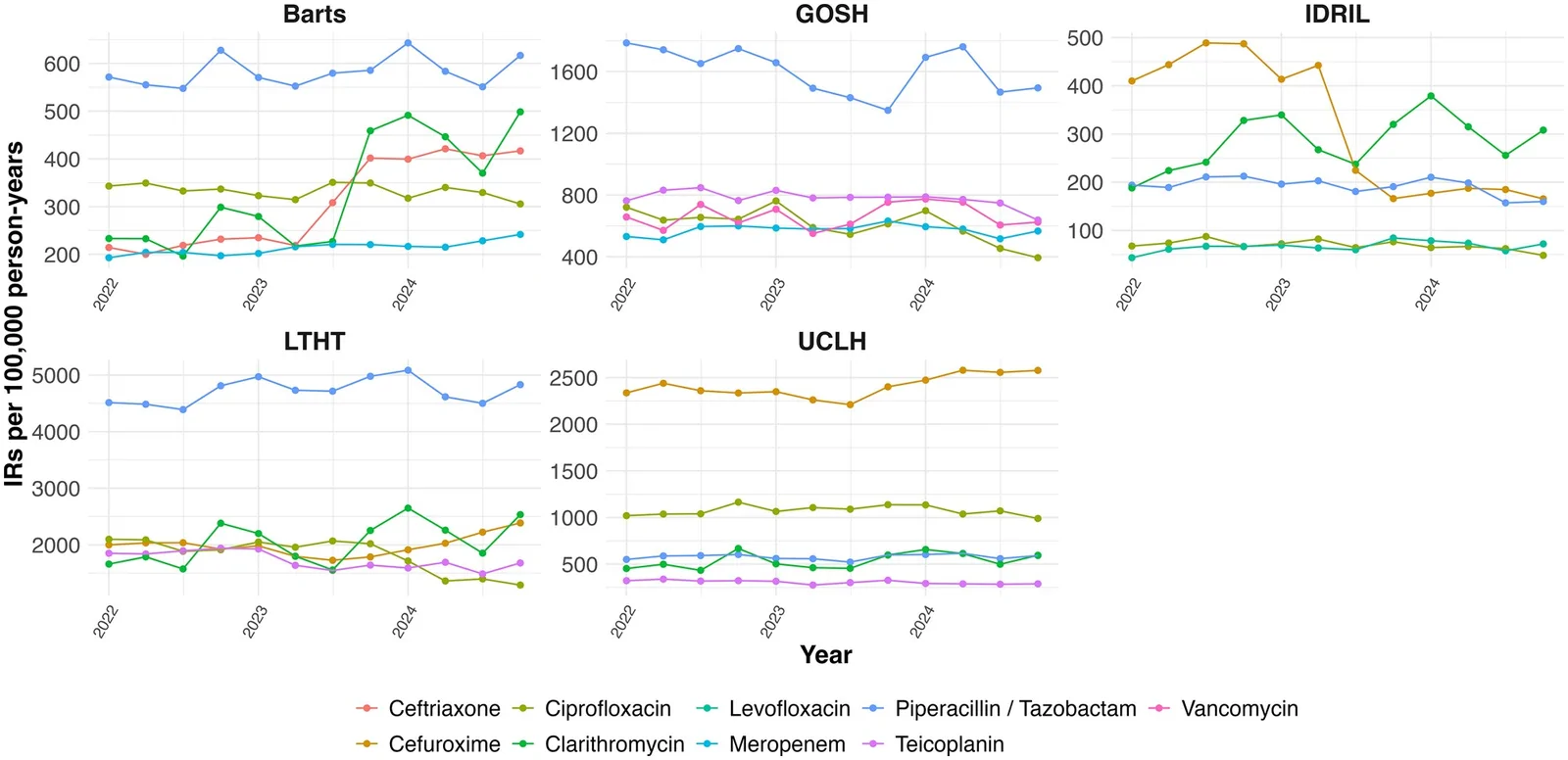
Quarterly crude IRs of the five most used Watch antibiotics across hospital databases.

In community care, IR displayed seasonality across all antibiotics, typically peaking in the third and fourth quarters of each year. Neomycin had the highest IR across the study period for CPRD Aurum (Fig. S1), increasing from 2183 (2168–2199) per 100 000 person years in Q1 2012–2322 (2307–2337) in Q1 2020. A decline in neomycin incidence was observed at the onset of the COVID-19 pandemic, falling to 1839 (1826–1853) in Q2 2020, before subsequently increasing to 2090 (2074–2106) in Q4 2024. In DataLoch, neomycin incidence declined between Q1 2012 and Q1 2020 [from 2464 (2404–2524) to 1982 (1935–2030)]. Further decline was then observed at the onset of the COVID-19 pandemic, falling to 1611 (1568–1654) in Q2 2020, followed by a gradual recovery [1744 (1701–1787) in Q4 2024].

Clarithromycin had the highest incidence across the study period for DataLoch; however, the IR declined significantly from 4883 (4799–4968) in Q1 2012–1914 (1868–1961) in Q1 2020. During the COVID-19 pandemic, clarithromycin incidence began to increase, eventually reaching 2374 (2324–2425) in Q2 2024. In contrast, IR of clarithromycin in CPRD Aurum remained relatively stable over time, although a noticeable spike was observed in Q4 2022.

Fusidate and erythromycin showed decreasing IR between Q1 2012 and Q1 2020 in both CPRD Aurum [from 2471 (2454–2487) to 593 (585–600), and from 1604 (1591–1617) to 440 (434–447) per 100 000 person-years, respectively] and DataLoch [from 3594 (3522–3668) to 2112 (2063–2161), and from 2428 (2369–2488) to 463 (441–487) per 100 000 person-years, respectively]. From 2020 onwards, IR remained stable.

There were some differences in IR between males and females for several of the top Watch antibiotics in both hospital and community care databases (Figs S2–S8). While both sexes followed similar trends, incidence was higher in females for neomycin, erythromycin, and fusidate in community care. There were no sex differences for clarithromycin in CPRD Aurum; however, incidence was higher in females for clarithromycin in DataLoch. In addition, there were no sex differences for ciprofloxacin in CPRD Aurum or for oxytetracycline in DataLoch. In hospital care, there were either no sex differences, or males had higher IR, with some exceptions, e.g. cefuroxime showed higher IR in females at LTHT and UCLH.

There were also some differences in IR between different age groups in community care (Figs S9–S14). For both CPRD Aurum and DataLoch, incidence of neomycin was highest in patients ≥65, and incidence of erythromycin was highest in children (patients under 18). However, for CPRD Aurum, incidence of clarithromycin use was highest in children, while in DataLoch, it was highest in patients over 65. However, all age groups typically followed the same trends over the study period.

In all hospitals except GOSH, incidence of the top five antibiotics is typically highest in patients ≥65, with some exceptions. In LTHT, incidence of cefuroxime was highest in patients 18–64 [overall incidence: 2819 (2759–2880)], followed by patients under 18 [1464 (1372–1560)]. However, in IDRIL and UCLH, overall incidence was highest in patients ≥65 [584 (570–599) and 3051 (2999–3104), respectively]. In addition, no significant difference was found in overall incidence of teicoplanin use in LTHT between the three age groups [0–17: 1691 (1592–1794), 18–64: 1414 (1372–1457), ≥65: 1908 (1851–1965)]; however, in UCLH, overall incidence was highest in the ≥65 group [463 (443–484)], and similar in the 0–17 and 18–64 groups [212 (192–232) and 203 (196–210), respectively].

### Prior use of Access and Watch antibiotics

Prior use of Access antibiotics within 14 days before Watch antibiotics showed some variation across databases, although there were some similarities (Fig. 2). In the hospital care databases, amoxicillin was one of the most frequently prescribed Access antibiotics prior to the Watch antibiotics piperacillin/tazobactam ranging from 15.3% in LTHT to 51.2% in Barts. In community care, amoxicillin was often the only Access antibiotic prescribed in more than 5% of cases before Watch antibiotic use. Distinct patterns were seen for GOSH, where amikacin was among the most prescribed prior Access antibiotics prior to Watch antibiotics ranging from 11.9% for piperacillin/tazobactam and 45.6% for vancomycin. At UCLH, trimethoprim and sulfamethoxazole were prescribed prior to Watch antibiotic, a pattern not seen in other hospital care databases, whereas gentamicin was prescribed prior to the top Watch antibiotics for IDRIL. For DataLoch, prior use of Access antibiotics was identified in fewer than 5% of users of the five most common Watch antibiotics and therefore was not presented in Fig. 2.

**Figure 2:**
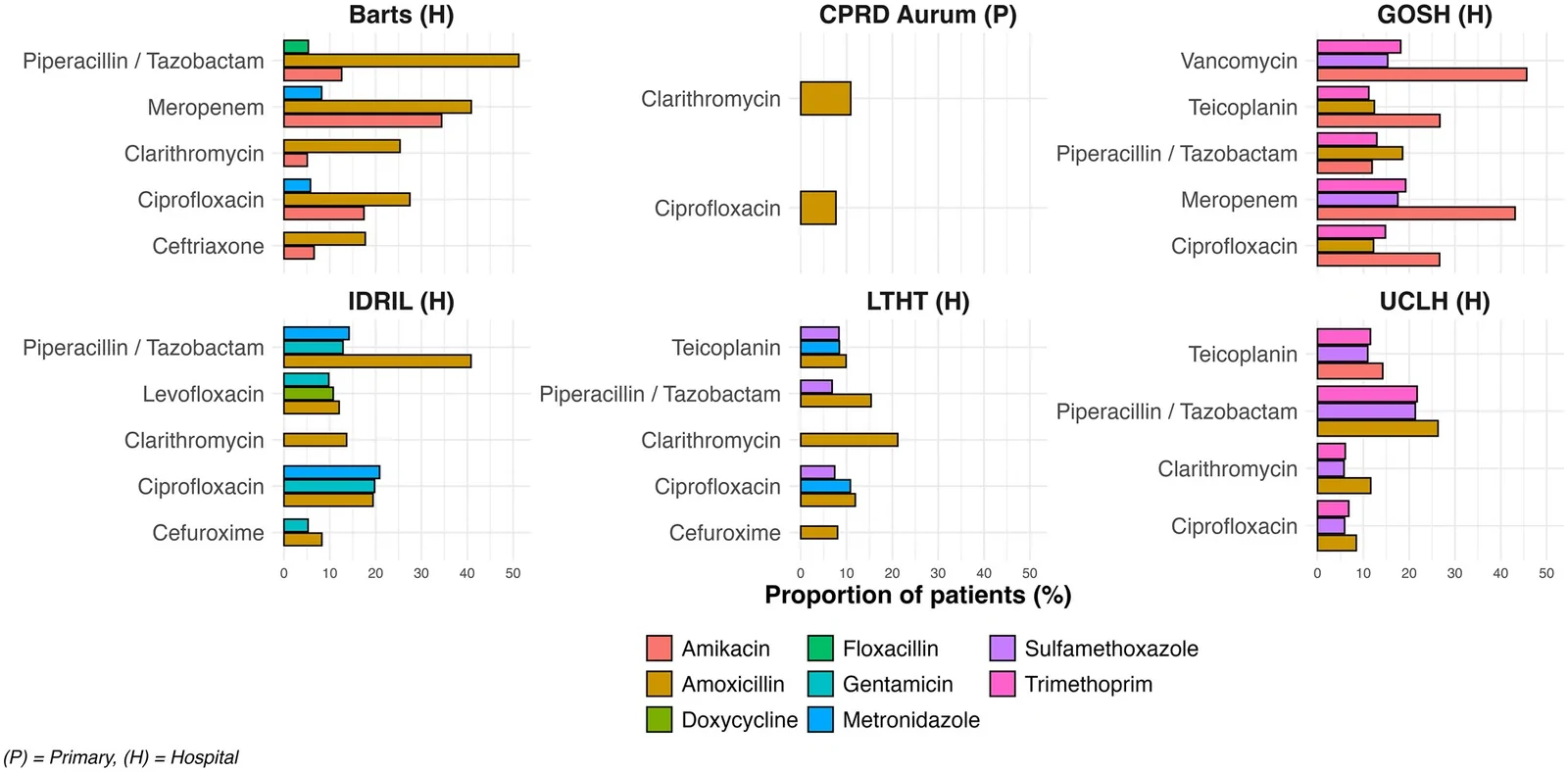
Use of Access antibiotics 14 days prior to use of a top five Watch antibiotic, stratified by database. Only includes Access antibiotics recorded in at least 5% of users.

Prior use of other Watch antibiotics also showed variation across databases (Fig. S15). Piperacillin/tazobactam was commonly given prior to other Watch antibiotics in hospital care. For example, in Barts and GOSH, approximately a third of patients that received meropenem had a record of piperacillin/tazobactam use in the 14 days prior (33.0% and 33.5%, respectively). Cefuroxime was also commonly used before ciprofloxacin in IDRIL, LTHT, and UCLH (18.5%, 10.1%, and 7.8%, respectively). For both CPRD Aurum and DataLoch, prior use of other Watch antibiotics was identified in fewer than 5% of users of the top five Watch antibiotics, therefore, were not presented in Fig. S15.

### Conditions of interest related to antibiotic treatment indications

There was a wide range of conditions of interest recorded alongside Watch antibiotic use, with differences between community and hospital care, as well as some differences between hospitals (Fig. 3).

**Figure 3:**
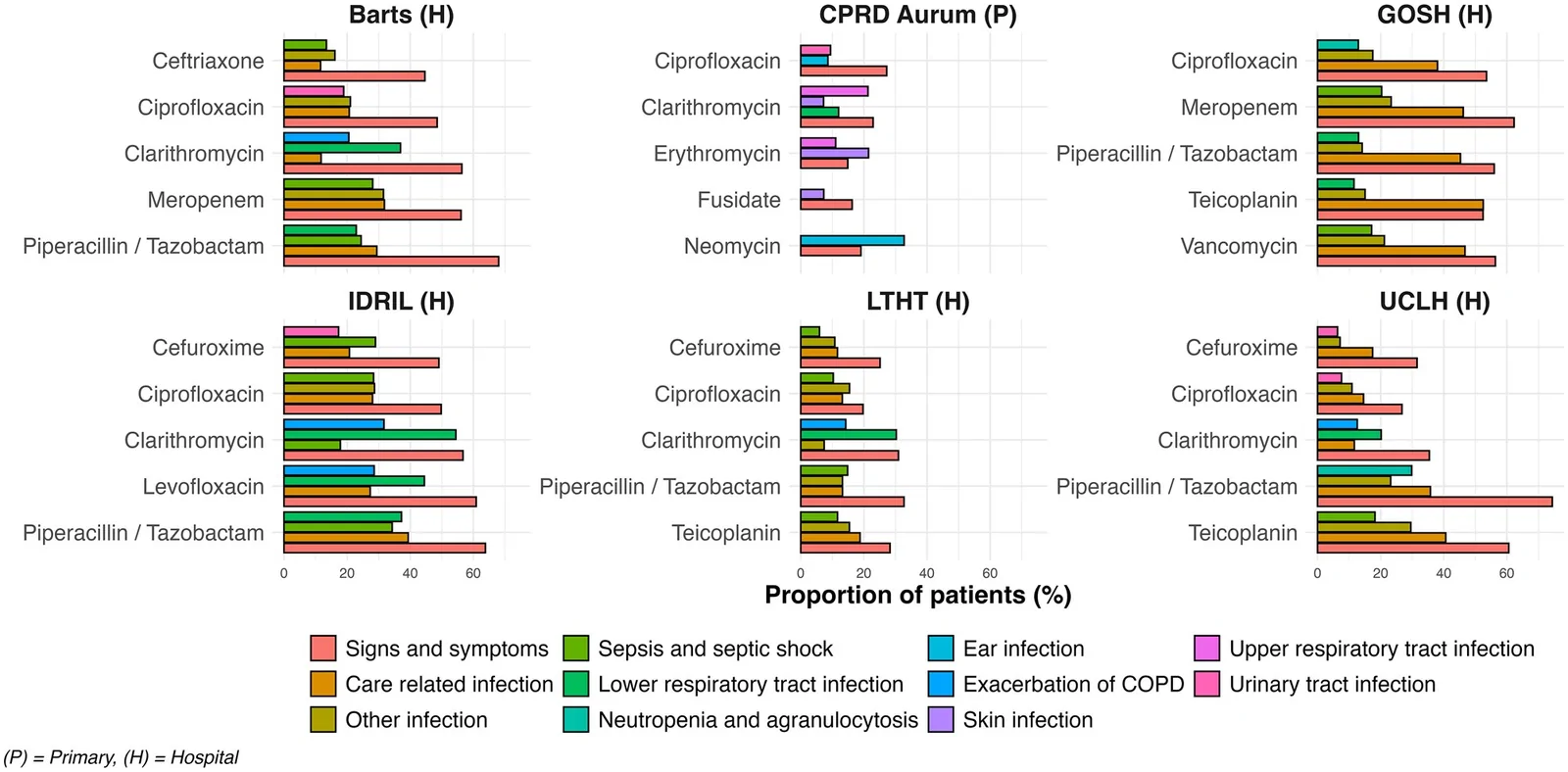
The four most frequent conditions of interest recorded in patients in the 14 days prior and 14 days after top five Watch antibiotics across databases. Only includes conditions recorded in at least 5% of users.

In all hospital care databases except GOSH, lower respiratory tract infections were commonly associated with clarithromycin use, ranging from 11.7% in UCLH to 54.4% in IDRIL with exacerbations of COPD also recorded around the time of use (12.6%–31.7%). For meropenem, Barts and GOSH showed similar patterns, with sepsis and septic shock (28.1% and 20.3%), care-related infections (31.8% and 46.2%), and other infections (31.5% and 23.3%) recorded. For ciprofloxacin, sepsis and septic shock were recorded in IDRIL and LTHT (28.4% and 10.3%) whereas Barts and UCLH recorded urinary tract infections (18.9% and 7.6%, respectively) on top of care-related infections and other infections. For GOSH, care-related infections were the most frequently recorded conditions, ranging from 38.0% for ciprofloxacin and 52.5% for teicoplanin. Care-related infections were also commonly recorded among the top Watch antibiotics for UCLH, ranging from 11.6% for clarithromycin to 40.6% for teicoplanin. In community care, CPRD Aurum showed ear infections recorded alongside neomycin (32.7%) while fusidate, erythromycin, and clarithromycin were predominately recorded alongside skin infections.

## Discussion

### Principal findings

We analysed over 13.8 million records of Watch antibiotic use from seven UK databases. Piperacillin/tazobactam appeared in the top five antibiotics at every hospital, although the associated conditions of interest varied: care-related infections were most common at Barts, GOSH, IDRIL, and UCLH, while sepsis and septic shock were the most common at LTHT. Ciprofloxacin also featured in the top five across all hospitals, with variation in associated conditions: it was linked to ‘other’ infections (site not specified) at Barts, IDRIL, and LTHT, and to care-related infections at GOSH and UCLH. A high proportion of ‘signs and symptoms’ were observed across all top five Watch antibiotics in hospital care.

Quarterly IRs of Watch antibiotic use and patient characteristics varied by hospital; however, higher incidence of Watch antibiotic use in male patients was consistently observed across all hospitals, in contrast to community care, where use was generally higher among female patients.

Although there was minimal overlap in the top five Watch antibiotics between community and hospital care, four of the top five antibiotics were the same in CPRD Aurum and DataLoch. Both databases also showed similar trends, with most Watch antibiotics declining or stable over the study period. However, for some antibiotics, incidence trends increased following the COVID-19 pandemic; however, they typically remained below prepandemic levels. Patient characteristics were also broadly consistent across both databases.

Conditions of interest recorded in the 14 days before and after in-hospital Watch antibiotic initiation were broadly consistent with the approved indications for use outlined in national guidelines. However, substantial variation was observed between hospitals. For example, piperacillin/tazobactam is approved for a wide range of indications, including sepsis, exacerbation of COPD, and infections in neutropenic patients [15]. While these conditions were commonly identified in our analysis, their frequency varied considerably across hospitals. Neutropenia and agranulocytosis ranged from 0% in LTHT to 29.8% in UCLH, while sepsis and septic shock ranged from 8.5% in GOSH to 34.3% in IDRIL, and exacerbation of COPD varied from 0.45% in GOSH to 22.8% in IDRIL. In contrast, the proportion of recorded conditions of interest was substantially lower in community care.

In both community and hospital care, many patients using Watch antibiotics had prior use of Access and/or other Watch antibiotics, although this was typically observed in fewer than half of patients.

### Comparison with other studies

Our findings align with previous UK and international research, demonstrating variability in antibiotic use across care settings and regions [16–20]. Variation in hospital care use is likely driven by stewardship protocols, local governance, and hospital-specific policies with some centres managing more resistant or complex infections [21]. Regional microbiological variation, including differences in bacterial strains and resistance may necessitate different prescribing/dispensing practices [22, 23]. Socio-demographics, health-seeking behaviour, comorbidity profiles, and differences between adult and paediatric populations further impacting use patterns [24, 25].

Differences between community and hospital care settings reflect the role of care setting in shaping prescribing/dispensing and stewardship priorities in the NHS. In community care, most uses relate to common and milder infections such as upper respiratory tract infections, often treated with Access list antibiotics [26]. In contrast, Watch antibiotics were more frequently used in hospitals to treat severe infections, surgical prophylaxis, or for patients requiring broader-spectrum coverage [10, 27]. The variation observed between hospitals likely reflects differences in specialist services and local antimicrobial prescribing guidelines, all of which may influence patterns of Watch antibiotic use.

The use of Watch list antibiotics should ideally follow appropriate first-line treatment, which are typically Access group antibiotics or in some cases other Watch antibiotics. We found that many patients had used Access antibiotics prior to initiation of a Watch antibiotic, suggesting escalation in line with clinical guidelines [10]. However, prior use of Access antibiotics was typically recorded in less than 50% of patients, suggesting Watch antibiotics were also commonly used as first-line treatment, possibly due to clinically assessed severity and/or bacterial resistance [28].

We observed higher Watch antibiotic use in female patients in community care, aligning with previous studies [29, 30]. This may reflect healthcare-seeking behaviours, with females being more likely to seek community care treatment [31]. In contrast, Watch antibiotic use was higher among male patients in hospital care. This may reflect differences in the severity or management of infections requiring hospital admission, although the reasons underlying this pattern warrant further investigation. Higher Watch antibiotic use in females in community care may also be explained by the higher rates of urinary tract infections (UTIs) among females, which often require antibiotic treatment [32]. Our results show that fosfomycin was the Watch antibiotic with the highest proportion of female users (81%–82%), which may be due to fosfomycin being the only Watch antibiotic recommended by NICE as second-line treatment for UTIs in nonpregnant females [8]. Further research is needed to explore the factors underlying sex differences in Watch antibiotic use and to better understand the role of specific indications, in shaping prescribing patterns.

The decline in antibiotic use in community care at the onset of the COVID-19 pandemic is consistent with findings from previous studies [33] and likely reflects the decrease in GP consultations following the introduction of lockdown measures [34]. The subsequent increase in the use of some Watch antibiotics may be partly due to the increase in remote consultations during the pandemic, with evidence suggesting that antibiotic prescribing rates are often higher in remote than in in-person consultations [35].

### Strengths and weaknesses of the study

A major strength of this study is the large-scale encompassing over 13.8 million patient records across multiple databases and care settings. This comprehensive approach enhances generalizability and offers a more complete picture of antibiotic use in the UK. In addition, this study provides unique insights that cannot be obtained using national data, as hospital-level information is not routinely collected. By using OMOP, it was possible to directly access data from care providers and enable distributed analytics without transferring patient-level data.

This study has several limitations. First, the indication of Watch antibiotic use was not recorded, therefore potential indications were identified based on conditions of interest recorded at the time of treatment initiation. Second, our analysis used the WHO 2023 AWaRe list, which may not fully reflect UK-specific adaptations [36]. Third, because community and hospital care data were generally not linked, we could not access prior community care records for hospital patients. Fourth, hospital-specific antibiotic guidelines were unavailable, preventing assessment of appropriate use against local recommendations. Fifth, due to limited data, it was not possible to reliably characterize trends in Watch antibiotic use over time in hospitals. Finally, due to data limitations, we analysed Watch antibiotics only at the ingredient level, without information on route or dose. This may affect interpretation, as different routes of administration for the same antibiotic can fall into different AWaRe categories.

### Implications for clinicians and policymakers

Understanding variations in antibiotic use, particularly those on the Watch list across care settings across the UK is critical for shaping targeted antimicrobial stewardship interventions, particularly with growing rates of AMR [37]. Our findings show variability across databases, particularly between hospital. This highlights opportunities to improve standardization of prescribing/dispensing practices and strengthen adherence to clinical guidelines. Unnecessary use due to lack of appropriate indication is associated with almost a third of global antibiotic consumption [38]. Therefore, our results give crucial insight into current use of Watch antibiotics in the UK and underscores the need for clear guidelines to ensure appropriate use in both community and hospital care. For clinicians, this study highlights increased awareness of resistance trends and the importance of accurate recording of infections or conditions that require antibiotic treatment. For policymakers, the study highlights the need for integrated stewardship programmes that span both community and hospital care.

### Unanswered questions and future research

Further research is needed to explore the underlying causes of observed variation in Watch antibiotic use, including regional factors, clinician preferences, and specific patient characteristics, as well as changes in global and national watchlists [39]. In addition, further research is needed to understand regional differences in dose and route of administration, and the impact this may have on AMR. Understanding how local governance structures, recommendations, and costings influence antibiotic selection in different regions could also help inform more effective stewardship strategies. Furthermore, qualitative research may also help explain decision-making processes that are not captured in structured data. Future studies should also incorporate microbiological data to assess appropriateness of antibiotic use and resistance patterns [40].

## Conclusion

This large, comprehensive study across community and hospital care settings in the UK provides valuable insights into Watch antibiotic use. However, there were marked differences between community and hospital care use. Our results highlight the complexity of antibiotic use across different settings and warrant further research to understand potential regional differences, local governance recommendations as well as the impact of different routes of administration.

## Supplementary Material

ckag198_Supplementary_Data

## Data Availability

Patient-level data cannot be shared without approval from data custodians owing to local information governance and data protection regulations. Analytical code for the study is freely available in a GitHub repository (https://github.com/heron-uk/HDRUK-01-001-Antibiotics). All study results can be found in the accompanying Shiny app (https://dpa-pde-oxford.shinyapps.io/heronAntibioticsStudy/). Key points Antibiotic prescribing/dispensing patterns can vary across care settings with most studies focusing on either community or hospital care in isolation. The WHO AWaRe classification highlights the need to monitor the use of Watch due to their higher potential to drive antimicrobial resistance. There is limited evidence across UK healthcare datasets examining real-world use of Watch list antibiotics and associated clinical indications. Variations in Watch list antibiotic prescribing/dispensing occur across community and hospital care settings. Conditions of interest of Watch list antibiotics patients were relatively consistent across general hospitals apart from paediatric hospital care and community care.
