# Supplementary material for: Patterns and potential indications of WHO AWaRe Watch antibiotic use across UK community and hospital care: a national observational study
Source: Eur J Public Health. 2026 Nov 3;36(6):ckag198. doi: 10.1093/eurpub/ckag198 (PMC13728849; doi:10.1093/eurpub/ckag198)
Supplement: Supplementary file 1 [file ckag198_supplementary_data.zip › ejph-2026-03-om-0332-File006.docx]

### Table S1: Database descriptions

| Database | Person count | Release date | Description |
| --- | --- | --- | --- |
| Barts | 3,234,945 | 30/08/2024 | Captures data on patients from hospitals within the trust, which includes The Royal London Hospital, Newham General Hospital, Whipps Cross Hospital, Mile End Hospital, and St Bartholomew’s Hospital. |
| CPRD Aurum | 50,370,041 | 20/09/2024 | The CPRD Aurum database contains longitudinal routinely-collected electronic health records (EHR data) from UK primary care practices using EMIS Web® general practice patient management software. |
| DataLoch | 1,753,685 | 05/08/2025 | The general population CDM contains person records drawn from the Lothian subsets of 4 national datasets: PHS Scottish Morbidity Records SMR00 (outpatient), SMR01 (inpatient), and SMR06 (cancer registry), and the PIS dataset for community prescribing information. These patients have been linked to historical coded data for hospitalisations, drug prescriptions, laboratory results, SIMD quintiles, GP data for BMI, alcohol consumption and smoking status, and deaths data from the National Records of Scotland. The dataset covers a geographical area in South East Scotland with a population of approximately 900k individuals. |
| GOSH | 203,386 | 05/11/2024 | GOSH is a specialist tertiary care paediatrics hospital in the UK receiving children with complex and/or rare conditions. A large proportion of the children that are seen at GOSH typically require long-term care and follow up at GOSH. This results in data collected during routine healthcare that are very rich in number of variables and detail. |
| IDRIL | 1,533,222 | 02/01/2025 | Captures data from Chorley and South Ribble Hospital, Royal Preston Hospital, and The Specialist Mobility and Rehabilitation Centre |
| LTHT | 1,486,578 | 08/05/2025 | Captures hospital data from patients who received care within the trust. |
| UCLH | 1,389,095 | 08/05/2025 | Captures hospital data from patients who received care within the trust, which includes University College Hospital, Royal National Throat, Nose and Ear Hospital, National Hospital for Neurology and Neurosurgery, and the UCH Macmillan Cancer Centre. |
