# Supplementary material for: Patterns and potential indications of WHO AWaRe Watch antibiotic use across UK community and hospital care: a national observational study
Source: Eur J Public Health. 2026 Nov 3;36(6):ckag198. doi: 10.1093/eurpub/ckag198 (PMC13728849; doi:10.1093/eurpub/ckag198)
Supplement: Supplementary file 1 [file ckag198_supplementary_data.zip › ejph-2026-03-om-0332-File007.docx]

### Table S2: Antibiotic from the Watch category of the WHO AWaRe classification (2023)

| Concept ID | Ingredient Name | Vocabulary |
| --- | --- | --- |
| 19101402 | arbekacin | RxNorm |
| 35198192 | aspoxicillin hydrate | RxNorm Extension |
| 1734104 | azithromycin | RxNorm |
| 19015123 | azlocillin | RxNorm |
| 36849346 | BEKANAMYCIN | RxNorm Extension |
| 35198093 | biapenem | RxNorm Extension |
| 1740546 | carbenicillin | RxNorm |
| 1768849 | cefaclor | RxNorm |
| 19070174 | cefamandole | RxNorm |
| 43009082 | cefbuperazone sodium | RxNorm Extension |
| 43009044 | cefcapene pivoxil hydrochloride hydrate | RxNorm Extension |
| 1796458 | cefdinir | RxNorm |
| 1747005 | cefditoren | RxNorm |
| 1748975 | cefepime | RxNorm |
| 19028241 | cefetamet | RxNorm |
| 1796435 | cefixime | RxNorm |
| 40798704 | Cefmenoxime | RxNorm Extension |
| 19072255 | cefmetazole | RxNorm |
| 43008993 | cefminox sodium | RxNorm Extension |
| 19028286 | cefodizime | RxNorm |
| 19072857 | cefonicid | RxNorm |
| 1773402 | cefoperazone | RxNorm |
| 19028288 | ceforanide | RxNorm |
| 36848891 | CEFOSELIS | RxNorm Extension |
| 1774470 | cefotaxime | RxNorm |
| 1774932 | cefotetan | RxNorm |
| 19051271 | cefotiam | RxNorm |
| 1775741 | cefoxitin | RxNorm |
| 35197975 | cefozopran hydrochloride | RxNorm Extension |
| 43009045 | cefpiramide sodium | RxNorm Extension |
| 19001904 | cefpirome | RxNorm |
| 1749008 | cefpodoxime | RxNorm |
| 1738366 | cefprozil | RxNorm |
| 19051345 | cefsulodin | RxNorm |
| 1776684 | ceftazidime | RxNorm |
| 35198137 | cefteram pivoxil | RxNorm Extension |
| 1749083 | ceftibuten | RxNorm |
| 1777254 | ceftizoxime | RxNorm |
| 1777806 | ceftriaxone | RxNorm |
| 1778162 | cefuroxime | RxNorm |
| 19095043 | chlortetracycline | RxNorm |
| 1797258 | cilastatin | RxNorm |
| 997496 | cinoxacin | RxNorm |
| 1797513 | ciprofloxacin | RxNorm |
| 1750500 | clarithromycin | RxNorm |
| 19047240 | clofoctol | RxNorm |
| 19047265 | clomocycline | RxNorm |
| 1592954 | delafloxacin | RxNorm |
| 1714527 | demeclocycline | RxNorm |
| 19023508 | dibekacin | RxNorm |
| 1790024 | dirithromycin | RxNorm |
| 1713905 | doripenem | RxNorm |
| 1743222 | enoxacin | RxNorm |
| 1717963 | ertapenem | RxNorm |
| 1746940 | erythromycin | RxNorm |
| 40239985 | fidaxomicin | RxNorm |
| 19050750 | fleroxacin | RxNorm |
| 43009087 | flomoxef sodium | RxNorm |
| 19064329 | flumequine | RxNorm |
| 36852196 | FLURITHROMYCIN | RxNorm Extension |
| 956653 | fosfomycin | RxNorm |
| 19010400 | fusidate | RxNorm |
| 35197938 | garenoxacin mesilate hydrate | RxNorm Extension |
| 1789276 | gatifloxacin | RxNorm |
| 1716721 | gemifloxacin | RxNorm |
| 1747032 | grepafloxacin | RxNorm |
| 1778262 | imipenem | RxNorm |
| 43009022 | isepamicin sulfate | RxNorm Extension |
| 19123240 | josamycin | RxNorm |
| 1784749 | kanamycin | RxNorm |
| 35834909 | lascufloxacin hydrochloride | RxNorm Extension |
| 1742253 | levofloxacin | RxNorm |
| 1790692 | lincomycin | RxNorm |
| 1707800 | lomefloxacin | RxNorm |
| 1708100 | loracarbef | RxNorm |
| 19092353 | lymecycline | RxNorm |
| 1709170 | meropenem | RxNorm |
| 19003644 | methacycline | RxNorm |
| 19007701 | mezlocillin | RxNorm |
| 19072089 | micronomicin | RxNorm |
| 19072122 | midecamycin | RxNorm |
| 1708880 | minocycline | RxNorm |
| 19009138 | miocamycin | RxNorm |
| 19126622 | moxalactam | RxNorm |
| 1716903 | moxifloxacin | RxNorm |
| 36878831 | nadifloxacin | RxNorm Extension |
| 36860386 | NEMONOXACIN | RxNorm Extension |
| 915981 | Neomycin | RxNorm |
| 19017585 | netilmicin | RxNorm |
| 1721543 | norfloxacin | RxNorm |
| 923081 | Ofloxacin | RxNorm |
| 19023254 | oleandomycin | RxNorm |
| 19129642 | oxolinic acid | RxNorm |
| 925952 | oxytetracycline | RxNorm |
| 35197853 | panipenem | RxNorm Extension |
| 35198003 | pazufloxacin mesilate | RxNorm Extension |
| 19027679 | pefloxacin | RxNorm |
| 19088795 | phenethicillin | RxNorm |
| 19010564 | pipemidate | RxNorm |
| 1746114 | piperacillin | RxNorm |
| 40799027 | Piromidic Acid | RxNorm Extension |
| 19125201 | pristinamycin | RxNorm |
| 35197897 | prulifloxacin | RxNorm Extension |
| 43009067 | ribostamycin sulfate | RxNorm Extension |
| 1777417 | rifabutin | RxNorm |
| 1763204 | rifampin | RxNorm |
| 19035924 | rifamycin SV | RxNorm |
| 1735947 | rifaximin | RxNorm |
| 35198144 | rokitamycin | RxNorm Extension |
| 19136024 | rolitetracycline | RxNorm |
| 19036545 | rosoxacin | RxNorm |
| 19063874 | roxithromycin | RxNorm |
| 35884386 | Rufloxacin | RxNorm Extension |
| 35200881 | sarecycline | RxNorm |
| 19136044 | sisomicin | RxNorm |
| 35198165 | sitafloxacin hydrate | RxNorm Extension |
| 36855357 | SOLITHROMYCIN | RxNorm Extension |
| 1733765 | sparfloxacin | RxNorm |
| 19070251 | spiramycin | RxNorm |
| 1836191 | streptomycin | RxNorm |
| 19136210 | streptozocin | RxNorm |
| 19000817 | sulbenicillin | RxNorm |
| 1741122 | tazobactam | RxNorm |
| 35198145 | tebipenem pivoxil | RxNorm Extension |
| 19078399 | teicoplanin | RxNorm |
| 1702911 | telithromycin | RxNorm |
| 19041153 | temafloxacin | RxNorm |
| 19100438 | temocillin | RxNorm |
| 1702364 | ticarcillin | RxNorm |
| 902722 | Tobramycin | RxNorm |
| 36857682 | TOSUFLOXACIN | RxNorm Extension |
| 19006043 | troleandomycin | RxNorm |
| 1712549 | trovafloxacin | RxNorm |
| 1707687 | vancomycin | RxNorm |
