# Supplementary material for: Patterns and potential indications of WHO AWaRe Watch antibiotic use across UK community and hospital care: a national observational study
Source: Eur J Public Health. 2026 Nov 3;36(6):ckag198. doi: 10.1093/eurpub/ckag198 (PMC13728849; doi:10.1093/eurpub/ckag198)
Supplement: Supplementary file 1 [file ckag198_supplementary_data.zip › ejph-2026-03-om-0332-File008.docx]

### Table S3: Antibiotics from the Access category of the WHO AWaRe classification (2023)

| Concept ID | Ingredient Name | Vocabulary |
| --- | --- | --- |
| 19123877 | amdinocillin | RxNorm |
| 19088223 | amdinocillin pivoxil | RxNorm |
| 1790868 | amikacin | RxNorm |
| 1713332 | amoxicillin | RxNorm |
| 1717327 | ampicillin | RxNorm |
| 19018516 | azidocillin | RxNorm |
| 1734205 | bacampicillin | RxNorm |
| 19018742 | brodimoprim | RxNorm |
| 40798709 | cefacetrile | RxNorm Extension |
| 1769535 | cefadroxil | RxNorm |
| 19070680 | cefatrizine | RxNorm |
| 40798700 | cefazedone | RxNorm Extension |
| 1771162 | cefazolin | RxNorm |
| 43009083 | cefroxadine | RxNorm Extension |
| 43008994 | ceftezole sodium | RxNorm Extension |
| 1786621 | cephalexin | RxNorm |
| 19052683 | cephaloridine | RxNorm |
| 19086759 | cephalothin | RxNorm |
| 19086790 | cephapirin | RxNorm |
| 1786842 | cephradine | RxNorm |
| 990069 | chloramphenicol | RxNorm |
| 997881 | clindamycin | RxNorm |
| 36863501 | clometocillin | RxNorm Extension |
| 1800835 | cloxacillin | RxNorm |
| 1724666 | dicloxacillin | RxNorm |
| 1738521 | doxycycline | RxNorm |
| 36851057 | epicillin | RxNorm Extension |
| 19054936 | floxacillin | RxNorm |
| 45892419 | gentamicin | RxNorm |
| 19069006 | hetacillin | RxNorm |
| 19072054 | methampicillin | RxNorm |
| 1361385 | methicillin | RxNorm |
| 1707164 | metronidazole | RxNorm |
| 1713930 | nafcillin | RxNorm |
| 40798981 | nifurtoinol | RxNorm Extension |
| 920293 | nitrofurantoin | RxNorm |
| 19024197 | ornidazole | RxNorm |
| 1724703 | oxacillin | RxNorm |
| 36857825 | penamecillin | RxNorm Extension |
| 1728416 | penicillin G | RxNorm |
| 1729720 | penicillin V | RxNorm |
| 19047071 | pivampicillin | RxNorm |
| 19096054 | propicillin | RxNorm |
| 19037983 | secnidazole | RxNorm |
| 1701651 | spectinomycin | RxNorm |
| 1836241 | sulbactam | RxNorm |
| 1836391 | sulfadiazine | RxNorm |
| 19000818 | sulfadimethoxine | RxNorm |
| 19136423 | sulfalene | RxNorm |
| 36856579 | sulfamazone | RxNorm Extension |
| 19136426 | sulfamerazine | RxNorm |
| 19136429 | sulfamethazine | RxNorm |
| 1036425 | sulfamethizole | RxNorm |
| 1836430 | sulfamethoxazole | RxNorm |
| 19000820 | sulfamethoxypyridazine | RxNorm |
| 36854873 | sulfametomidine | RxNorm Extension |
| 40799118 | sulfametoxydiazine | RxNorm Extension |
| 19040624 | sulfametrole | RxNorm |
| 19136481 | sulfamoxole | RxNorm |
| 1036475 | sulfanilamide | RxNorm |
| 40799120 | sulfaperin | RxNorm Extension |
| 40799121 | sulfaphenazole | RxNorm Extension |
| 19136493 | sulfapyridine | RxNorm |
| 1036487 | sulfathiazole | RxNorm |
| 19000821 | sulfisomidine | RxNorm |
| 1836503 | sulfisoxazole | RxNorm |
| 43009009 | sultamicillin | RxNorm Extension |
| 19002077 | talampicillin | RxNorm |
| 1836948 | tetracycline | RxNorm |
| 19137362 | thiamphenicol | RxNorm |
| 1702559 | tinidazole | RxNorm |
| 1705674 | trimethoprim | RxNorm |
