# Supplementary material for: Patterns and potential indications of WHO AWaRe Watch antibiotic use across UK community and hospital care: a national observational study
Source: Eur J Public Health. 2026 Nov 3;36(6):ckag198. doi: 10.1093/eurpub/ckag198 (PMC13728849; doi:10.1093/eurpub/ckag198)
Supplement: Supplementary file 1 [file ckag198_supplementary_data.zip › ejph-2026-03-om-0332-File009.docx]

### Table S4: Conditions of interest and their ancestor concepts.

| Condition of Interest | Ancestor Concept IDs | Ancestor Concept Names | Notes |
| --- | --- | --- | --- |
| Cardiac infection | 314383 | Myocarditis |  |
|  | 4138837 | Pericarditis |  |
|  | 441589 | Endocarditis |  |
| Care-related infection | 4193161 | Disorder following clinical procedure |  |
|  | 318445 | Post cardiac operation functional disturbance |  |
|  | 4123283 | Disorder of stoma |  |
|  | 43021974 | Complication associated with device |  |
|  | 4201387 | Tracheostomy present |  |
|  | 442019 | Complication of procedure |  |
| Ear infection | 4044878 | Infection of ear |  |
|  | 380731 | Otitis externa |  |
| Exacerbation of COPD | 255573 | Chronic obstructive pulmonary disease |  |
|  | 257004 | Acute exacerbation of chronic obstructive pulmonary disease |  |
| Exacerbation of cystic fibrosis | 441267 | Cystic fibrosis |  |
|  | 44808532 | Exacerbation of cystic fibrosis |  |
| Eye infection | 4134613 | Eye infection |  |
|  | 37160823 | Infection of eyelid caused by Mycobacterium leprae |  |
| GI infection | 37396146 | Gastrointestinal infection |  |
|  | 44783254 | Infection of masticator space |  |
|  | 198678 | Intestinal infectious disease |  |
|  | 198337 | Infectious diarrheal disease |  |
| Infection caused by antimicrobial resistant bacteria | 4249827 | Infection caused by antimicrobial resistant bacteria |  |
|  | 37017452 | Drug resistance to antibacterial agent |  |
|  | 44806682 | Infection resistant to multiple antibiotics |  |
| Lower respiratory tract infection | 4175297 | Lower respiratory tract infection |  |
|  | 4133224 | Lobar pneumonia |  |
|  | 618954 | Exacerbation of bronchiectasis caused by infection |  |
| Neutropenia and agranulocytosis | 320073 | Neutropenia |  |
|  | 440689 | Agranulocytosis |  |
| Other infection | 432545 | Bacterial infectious disease | Excluding concepts found in other conditions of interest |
| Sepsis and septic shock | 132797 | Sepsis |  |
|  | 196236 | Septic shock |  |
| Signs and symptoms | 201965 | Shock |  |
|  | 437663 | Fever |  |
|  | 254761 | Cough |  |
|  | 4305080 | Abnormal breathing |  |
|  | 31967 | Nausea |  |
|  | 441408 | Vomiting |  |
|  | 196523 | Diarrhoea |  |
|  | 254061 | Pleural effusion |  |
|  | 197672 | Urinary incontinence |  |
|  | 4103189 | Finding of heart rate | Excluding normal heart rate findings |
|  | 373995 | Delirium |  |
|  | 433595 | Oedema |  |
|  | 321689 | Apnoea |  |
|  | 253321 | Stridor |  |
|  | 200528 | Ascites |  |
|  | 435517 | Acidosis |  |
|  | 4128820 | Feeding finding |  |
|  | 435515 | Hypo-osmolality and or hyponatremia |  |
|  | 4214962 | Blood pressure finding | Excluding normal blood pressure findings |
|  | 44784217 | Cardiac arrhythmia |  |
| Skin infection | 201093 | Infection of skin and/or subcutaneous tissue |  |
|  | 141095 | Acne |  |
| Upper respiratory tract infection | 4181583 | Upper respiratory infection |  |
|  | 4234533 | Tonsillitis |  |
| Urinary tract infection | 81902 | Urinary tract infectious disease |  |
