# Supplementary material for: Patterns and potential indications of WHO AWaRe Watch antibiotic use across UK community and hospital care: a national observational study
Source: Eur J Public Health. 2026 Nov 3;36(6):ckag198. doi: 10.1093/eurpub/ckag198 (PMC13728849; doi:10.1093/eurpub/ckag198)
Supplement: Supplementary file 1 [file ckag198_supplementary_data.zip › ejph-2026-03-om-0332-File010.docx]

### Table S5: Patient characteristics for the top five antibiotics in Barts

| **Variable name** | **Variable level** | **Estimate name** | **Piperacillin / Tazobactam** | **Clarithromycin** | **Ciprofloxacin** | **Ceftriaxone** | **Meropenem** |
| --- | --- | --- | --- | --- | --- | --- | --- |
| Number  records |  | N | 19,976 | 11,794 | 11,359 | 10,841 | 7,417 |
| Number  subjects |  | N | 16,392 | 10,562 | 9,761 | 10,250 | 6,276 |
| Cohort start  date |  | Range | 2022-01-01  to  2025-05-04 | 2022-01-01  to  2025-05-03 | 2022-01-01  to  2025-05-04 | 2022-01-01  to  2025-05-04 | 2022-01-01  to  2025-05-03 |
| Age |  | Median  [Q25 –  Q75] | 65 [50 - 77] | 65 [44 - 79] | 62 [43 - 76] | 38 [19 - 60] | 61 [44 - 74] |
|  |  | Range | 0 to 113 | 0 to 114 | 0 to 113 | 0 to 114 | 0 to 102 |
| Sex | Female | N (%) | 8,554  (42.82%) | 5,981  (50.71%) | 5,644  (49.69%) | 4,993  (46.06%) | 3,212  (43.31%) |
|  | Male | N (%) | 11,422  (57.18%) | 5,813  (49.29%) | 5,715  (50.31%) | 5,848  (53.94%) | 4,205  (56.69%) |
