# Supplementary material for: Patterns and potential indications of WHO AWaRe Watch antibiotic use across UK community and hospital care: a national observational study
Source: Eur J Public Health. 2026 Nov 3;36(6):ckag198. doi: 10.1093/eurpub/ckag198 (PMC13728849; doi:10.1093/eurpub/ckag198)
Supplement: Supplementary file 1 [file ckag198_supplementary_data.zip › ejph-2026-03-om-0332-File011.docx]

### Table S6: Patient characteristics for the top five antibiotics in GOSH

| **Variable name** | **Variable level** | **Estimate name** | **Piperacillin / Tazobactam** | **Teicoplanin** | **Vancomycin** | **Ciprofloxacin** | **Meropenem** |
| --- | --- | --- | --- | --- | --- | --- | --- |
| Number  records |  | N | 4,227 | 2,025 | 1,757 | 1,571 | 1,527 |
| Number  subjects |  | N | 3,143 | 1,635 | 1,367 | 1,204 | 1,173 |
| Cohort  start date |  | Range | 2022-01-01  to  2025-02-17 | 2022-01-04  to  2025-02-17 | 2022-01-02  to  2025-02-16 | 2022-01-02  to  2025-02-17 | 2022-01-03  to  2025-02-17 |
| Age |  | Median  [Q25 –  Q75] | 2 [0 - 8] | 4 [0 - 11] | 1 [0 - 7] | 4 [1 - 10] | 2 [0 - 7] |
|  |  | Range | 0 to 17 | 0 to 17 | 0 to 17 | 0 to 17 | 0 to 17 |
| Sex | Female | N (%) | 1,772  (41.92%) | 879  (43.41%) | 757  (43.08%) | 692  (44.05%) | 644  (42.17%) |
|  | Male | N (%) | 2,455  (58.08%) | 1,146  (56.59%) | 1,000  (56.92%) | 879  (55.95%) | 883  (57.83%) |
