# Supplementary material for: Patterns and potential indications of WHO AWaRe Watch antibiotic use across UK community and hospital care: a national observational study
Source: Eur J Public Health. 2026 Nov 3;36(6):ckag198. doi: 10.1093/eurpub/ckag198 (PMC13728849; doi:10.1093/eurpub/ckag198)
Supplement: Supplementary file 1 [file ckag198_supplementary_data.zip › ejph-2026-03-om-0332-File012.docx]

### Table S7: Patient characteristics for the top five antibiotics in IDRIL

| **Variable name** | **Variable level** | **Estimate name** | **Cefuroxime** | **Clarithromycin** | **Piperacillin / Tazobactam** | **Ciprofloxacin** | **Levofloxacin** |
| --- | --- | --- | --- | --- | --- | --- | --- |
| Number  records |  | N | 10,801 | 9,870 | 6,642 | 2,400 | 2,314 |
| Number  subjects |  | N | 9,634 | 8,281 | 5,857 | 2,177 | 1,984 |
| Cohort  start date |  | Range | 2022-01-01  to  2024-12-30 | 2022-01-01  to  2025-01-01 | 2022-01-01  to  2024-12-31 | 2022-01-01  to  2024-12-29 | 2022-01-02  to  2024-12-27 |
| Age |  | Median  [Q25 –  Q75] | 69 [50 - 80] | 75 [62 - 84] | 72 [60 - 81] | 68 [55 - 78] | 76 [62 - 84] |
|  |  | Range | 0 to 104 | 0 to 104 | 0 to 104 | 1 to 102 | 7 to 101 |
| Sex | Female | N (%) | 5,475  (50.69%) | 4,655  (47.16%) | 2,843  (42.80%) | 1,184  (49.33%) | 1,222  (52.81%) |
|  | Male | N (%) | 5,326  (49.31%) | 5,215  (52.84%) | 3,799  (57.20%) | 1,216  (50.67%) | 1,092  (47.19%) |
