# Supplementary material for: Patterns and potential indications of WHO AWaRe Watch antibiotic use across UK community and hospital care: a national observational study
Source: Eur J Public Health. 2026 Nov 3;36(6):ckag198. doi: 10.1093/eurpub/ckag198 (PMC13728849; doi:10.1093/eurpub/ckag198)
Supplement: Supplementary file 1 [file ckag198_supplementary_data.zip › ejph-2026-03-om-0332-File013.docx]

### Table S8: Patient characteristics for the top five antibiotics in LTHT

| **Variable name** | **Variable level** | **Estimate name** | **Piperacillin / Tazobactam** | **Clarithromycin** | **Cefuroxime** | **Ciprofloxacin** | **Teicoplanin** |
| --- | --- | --- | --- | --- | --- | --- | --- |
| Number  records |  | N | 26,365 | 11,567 | 11,270 | 10,043 | 9,608 |
| Number  subjects |  | N | 20,460 | 10,318 | 10,412 | 8,661 | 8,401 |
| Cohort  start date |  | Range | 2022-01-01  to  2025-03-18 | 2022-01-01  to  2025-03-19 | 2022-01-01  to  2025-03-19 | 2022-01-01  to  2025-03-19 | 2022-01-01  to  2025-03-18 |
| Age |  | Median  [Q25 –  Q75] | 72 [57 - 81] | 72 [54 - 82] | 40 [28 - 60] | 64 [44 - 77] | 61 [42 - 75] |
|  |  | Range | 0 to 108 | 0 to 108 | 0 to 100 | 0 to 102 | 0 to 105 |
| Sex | Female | N (%) | 11,562  (43.85%) | 5,522  (47.74%) | 7,178  (63.69%) | 4,845  (48.24%) | 4,569  (47.55%) |
|  | Male | N (%) | 14,803  (56.15%) | 6,045  (52.26%) | 4,092  (36.31%) | 5,198  (51.76%) | 5,039  (52.45%) |
