# Supplementary material for: Patterns and potential indications of WHO AWaRe Watch antibiotic use across UK community and hospital care: a national observational study
Source: Eur J Public Health. 2026 Nov 3;36(6):ckag198. doi: 10.1093/eurpub/ckag198 (PMC13728849; doi:10.1093/eurpub/ckag198)
Supplement: Supplementary file 1 [file ckag198_supplementary_data.zip › ejph-2026-03-om-0332-File014.docx]

### Table S9: Patient characteristics for the top five antibiotics in UCLH

| **Variable name** | **Variable level** | **Estimate name** | **Cefuroxime** | **Ciprofloxacin** | **Piperacillin / Tazobactam** | **Clarithromycin** | **Teicoplanin** |
| --- | --- | --- | --- | --- | --- | --- | --- |
| Number  records |  | N | 44,228 | 19,251 | 10,450 | 9,842 | 5,547 |
| Number  subjects |  | N | 39,445 | 16,506 | 7,995 | 8,759 | 4,887 |
| Cohort  start date |  | Range | 2022-01-01  to  2025-05-12 | 2022-01-01  to  2025-05-12 | 2022-01-01  to  2025-05-09 | 2022-01-01  to  2025-05-12 | 2022-01-01  to  2025-05-09 |
| Age |  | Median  [Q25 –  Q75] | 50 [35 - 67] | 52 [32 - 67] | 59 [41 - 71] | 57 [36 - 71] | 57 [37 - 70] |
|  |  | Range | 0 to 107 | 0 to 101 | 0 to 104 | 0 to 105 | 0 to 104 |
| Sex | Female | N (%) | 27,009  (61.07%) | 9,688  (50.32%) | 4,541  (43.45%) | 5,175  (52.58%) | 2,995  (53.99%) |
|  | Male | N (%) | 17,219  (38.93%) | 9,563  (49.68%) | 5,909  (56.55%) | 4,667  (47.42%) | 2,552  (46.01%) |
