# Supplementary material for: Patterns and potential indications of WHO AWaRe Watch antibiotic use across UK community and hospital care: a national observational study
Source: Eur J Public Health. 2026 Nov 3;36(6):ckag198. doi: 10.1093/eurpub/ckag198 (PMC13728849; doi:10.1093/eurpub/ckag198)
Supplement: Supplementary file 1 [file ckag198_supplementary_data.zip › ejph-2026-03-om-0332-File015.docx]

### Table S10: Patient characteristics for the top five antibiotics in CPRD Aurum

| **Variable name** | **Variable level** | **Estimate name** | **Neomycin** | **Fusidate** | **Ciprofloxacin** | **Erythromycin** | **Clarithromycin** |
| --- | --- | --- | --- | --- | --- | --- | --- |
| Number  records |  | N | 4,477,598 | 2,029,304 | 1,561,067 | 1,295,225 | 769,070 |
| Number  subjects |  | N | 2,627,866 | 1,446,531 | 1,019,792 | 722,514 | 537,083 |
| Cohort  start date |  | Range | 2012-01-01  to  2024-12-18 | 2012-01-01  to  2024-12-17 | 2012-01-01   to  2024-12-18 | 2012-01-01   to  2024-12-18 | 2012-01-01   to  2024-12-17 |
| Age |  | Median  [Q25 –  Q75] | 49 [28 - 66] | 44 [11 - 68] | 59 [41 - 74] | 16 [6 - 31] | 6 [3 - 13] |
|  |  | Range | 0 to 111 | 0 to 111 | 0 to 109 | 0 to 111 | 0 to 112 |
| Sex | Female | N (%) | 2,535,489 (56.63%) | 1,114,817  (54.94%) | 769,680  (49.30%) | 805,653  (62.20%) | 399,320  (51.92%) |
|  | Male | N (%) | 1,942,109 (43.37%) | 914,487 (45.06%) | 791,387  (50.70%) | 489,572   (37.80%) | 369,750  (48.08%) |
