# Supplementary material for: Patterns and potential indications of WHO AWaRe Watch antibiotic use across UK community and hospital care: a national observational study
Source: Eur J Public Health. 2026 Nov 3;36(6):ckag198. doi: 10.1093/eurpub/ckag198 (PMC13728849; doi:10.1093/eurpub/ckag198)
Supplement: Supplementary file 1 [file ckag198_supplementary_data.zip › ejph-2026-03-om-0332-File016.docx]

### Table S11: Patient characteristics for the top five antibiotics in DataLoch

| **Variable name** | **Variable level** | **Estimate name** | **Clarithromycin** | **Fusidate** | **Neomycin** | **Oxytetracycline** | **Erythromycin** |
| --- | --- | --- | --- | --- | --- | --- | --- |
| Number  records |  | N | 394,394 | 390,456 | 353,001 | 178,584 | 136,037 |
| Number  subjects |  | N | 209,695 | 237,399 | 173,806 | 65,386 | 60,640 |
| Cohort  start date |  | Range | 2012-01-31   to  2025-03-31 | 2012-01-31  to  2025-03-31 | 2012-01-31 to  2025-03-31 | 2012-01-31   to  2025-03-31 | 2012-01-31  to  2025-03-31 |
| Age |  | Median  [Q25 –  Q75] | 50 [31 - 66] | 38 [14 - 62] | 51 [31 - 66] | 50 [31 - 65] | 30 [18 - 48] |
|  |  | Range | 0 to 108 | 0 to 107 | 0 to 105 | 0 to 105 | 0 to 107 |
| Sex | Female | N (%) | 244,700  (62.04%) | 215,991   (55.32%) | 206,292  (58.44%) | 92,335  (51.70%) | 87,630  (64.42%) |
|  | Male | N (%) | 149,694  (37.96%) | 174,465  (44.68%) | 146,709  (41.56%) | 86,249  (48.30%) | 48,407  (35.58%) |
